# Intergenerational Transmission of Relational Styles: Current Considerations

**DOI:** 10.3389/fpsyg.2021.672961

**Published:** 2021-09-30

**Authors:** Federica Taccini, Alessandro Alberto Rossi, Stefania Mannarini

**Affiliations:** ^1^Department of Philosophy, Sociology, Education, and Applied Psychology, Section of Applied Psychology, University of Padova, Padova, Italy; ^2^Interdepartmental Center for Family Research, University of Padova, Padova, Italy

**Keywords:** relational competence, relational style, intergenerational transmission, violence, caregivers, clinical psychology, psychodynamic psychology

## Introduction

In recent decades, the intergenerational transmission of some psychological issues and difficulties such as trauma has attracted the attention of many researchers due to the process for which these difficulties “*were carried over from one generation to the next*” (Kellermann, 2001, p. 257). In this regard, several studies were conducted within the spectrum of trauma in war-related contexts, highlighting the aforementioned intergenerational transmission, for example, from the victims of the holocaust to their children and grandchildren (Fonagy, 1999; Kellermann, 2001; Yehuda et al., 2008; Shmotkin et al., 2011; Shrira et al., 2011; Roitman, 2017).

Recently, numerous studies were developed to investigate this complex process also in the non-war-related traumatic experiences (Schwerdtfeger et al., 2013; Lehrner and Yehuda, 2018; Steketee et al., 2019) such as the intergenerational transmission of violence. For example, the literature underlines that the exposure of children to violence, together with several other factors (e.g., lack of education and poverty), may contribute to their enactment of similar (active or passive) conducts in their interactions with others (Kwong et al., 2003; Franklin and Kercher, 2012; Savage et al., 2014; Russell et al., 2020).

The literature suggested the possibility of the intergenerational transmission of violence (Black et al., 2010; Lünnemann et al., 2019). In fact, children who have been exposed to, or witnessed, parental violence seem to be at higher risk of exhibiting violent behaviors toward both their partner and descendants in the future (Rikić et al., 2017). In a 20-year prospective study, Ehrensaft et al. (2003) showed that witnessing domestic violence between caregivers (e.g., parents and guardians) may contribute to adopting similar (dysfunctional) ways of managing conflicts with loved ones in adulthood. Also, Assink et al. (2018) pointed out that victims of child abuse and neglect are three times more likely to perpetrate similar conduct in their future relationships. Early experiences of an abusive environment also seem to contribute to the victimization by violent partners (Stith et al., 2000; Tyler et al., 2011; Sutton et al., 2014).

This intergenerational transmission of violence could rely on different mechanisms, but among them, two main processes seem to be relevant. On the one hand, through the processes of imitating, modeling, and made active experiences, children exposed to violence in their family context could learn that violence is a feature of intimate relationships (Festinger and Carlsmith, 1959; Bandura, 1977, 1979; Simons et al., 2012). These children learned from their violent family environment that harsh parenting, violence, and aggression are the normal aspects of (intimate) relationships (Sellers et al., 2005; Simons et al., 2012), leading to relationships in adulthood that could be characterized by violence (Sellers et al., 2005; Simons et al., 2012). On the other hand, the experience of unreliable, unsupportive, and violent caregivers may contribute to the development of an insecure attachment of children (Dutton, 1999; Gustafsson et al., 2017; Velotti et al., 2018) which seems to represent a risk factor for being victim or perpetrator of domestic abuse (Yarkovsky and Timmons Fritz, 2014; Velotti et al., 2018).

In addition, it may also be possible that caregivers themselves had been traumatized by the dysfunctional relational styles of their own parents and that this traumatic experience remains unelaborated (Cusinato, 2013; Rikić et al., 2017). Similarly, the unresolved traumatic experiences of parents may be projected to and then internalized by their children who may be at risk to manifest them in their own future relationships (Fonagy, 1999; Roitman, 2017; Stob et al., 2020). Therefore, the experience of children on their parents as not being secure emotional bases may affect the future definition of the Self of children and their own attachment (Giladi and Bell, 2013; Mannarini et al., 2017; Roitman, 2017; Dashorst et al., 2019; Settineri et al., 2019). It is important to stress out that several protective factors may contribute to breaking off this cycle of intergenerational transmission of violence. In this regard, Tracy et al. (2018) investigated mistreatment and violence throughout three generations. Their results show the protective role of social support on subsequent violence, depending on the kind of violence history and timing (Tracy et al., 2018). Furthermore, higher socioeconomic status, positive relationships with the offspring, higher levels of satisfaction with parenthood, and functional interpersonal relationships in adulthood were found to mitigate this intergenerational transmission (McClellan and Killeen, 2000; Tracy et al., 2018; Langevin et al., 2019).

## The Relational Competence Theory and Relational Styles

Relational competence theory (RCT) (L'Abate, 2005) has been developed with the aim to understand the relational competence of individuals in intimate relationships, expanding previous theories by taking into account both relational and contextual features in the development of an individual (L'Abate, 1997). In fact, according to RCT, relational competence “*could be even defined as a synonym of personality*” (Cusinato, 2013, p. 6) since it corresponds to the overall interpersonal abilities of people and relational styles that develop during their lifetime throughout the different contexts they experience.

Some of the innovative aspects of RCT, which make it an object of clinical and research interest, are as follows: first, it aims to understand, anticipate, and improve the relational competence and styles of individuals (L'Abate, 2005). Second, it theorizes personality as having a relational nature: in fact, individuals develop their own personality through the interactions with intimate ones and throughout all the contexts they experience (Colesso, 2006a). Third, according to this theorization, intimate relationships are defined as corresponding to all the stable, interdependent, and committed relationships, regardless of the biological linkages (Cusinato and Colesso, 2009; L'Abate et al., 2010; Cusinato, 2013). This makes the RCT very useful in a dynamic and ever-changing society similar to ours. In addition, the RCT aims to accomplish three functions, “*namely, understanding, predicting, and controlling individuals in their intimate relationships and other settings*” (L'Abate, 2005, p. 14). Moreover, the RCT attempted to integrate both the categorical and dimensional approaches of relational styles (L'Abate et al., 2010). In fact, according to the RCT, intimate relationships are characterized by three different categories of styles, namely, the abusive-apathetic style (AA), the reactive-repetitive (RR) one, and the creative-conductive (CC) style, i.e., each person may show one of them as the main way of interacting with others (L'abate, 1983; L'Abate, 2005; L'abate and Cusinato, 2007). Each style ranges along a dimensional continuum from lower to higher levels, and these styles are separate and not mutually exclusive dimensional categories. In other words, everyone can have different levels of the three different styles, simultaneously. The AA could be considered as a dysfunctional relational style characterized by a tendency either to verbal or physical aggression or to deal with situations in incompetent and inconsistent ways. People with higher levels of AA style may tend to establish abusive and violent (i.e., abusive) and/or neglecting (i.e., apathetic) relationships. Concerning the second one, people with higher levels of RR style may tend to react immediately or in a withdrawing way to interactive dynamics (i.e., reactive) with the unconscious objective to maintain the relational interaction unchanged (i.e., repetitive). Therefore, these people may have difficulties in establishing intimate relationships. Regarding the third one, people with higher levels of CC style are more prone to look inward and take care of themselves and others (i.e., creativity). At the same time, they show abilities of being flexible and assertive with others in a constructive way (i.e., conductivity). Therefore, people with higher levels of this style may be able to establish intimate relationships in a functional way (Colesso, 2006b; L'Abate, 2008; L'Abate et al., 2010).

Some studies have supported this theory (L'Abate and Wagner, 1988; L'Abate, 2005, 2008, 2009, 2010; L'Abate et al., 2010; Gianesini, 2012; Cusinato, 2013; Colesso et al., 2018). For example, Gianesini (2012) has shown that the relational competence of parents was associated with parenting styles: parents adopting functional parenting style seem to present the functional relational competence also, and *vice versa*. In this regard, Colesso et al. (2018) have displayed a possible relationship between the alexithymic difficulties of a group of parents whose children present eating disorders and their relational competence. Further research is needed on the RCT to the end of a stronger empirical validation of the theory itself (L'Abate, 2008; L'Abate et al., 2010).

## Intergenerational Transmission of Relational Styles

Moreover, according to L'Abate (2005), relational styles may be transmitted across generations. Specifically, L'Abate (2005) defined intergenerational transmission as the “*transmission of particular relationship styles or symptoms from one generation to another, including the present situation in the family of procreation*” (L'Abate, 2005, p. 125). Therefore, according to RCT, the relational styles experienced with caregivers seem to influence the relational styles of children in adult relationships (L'Abate, 2005). Hence, there may be a positive side of the coin of this intergenerational transmission. In fact, the experience of caregivers with high levels of a supportive relational style (i.e., CC) may facilitate the manifestation of children of such functional styles (Cusinato, 2013). Experiencing a CC style in previous interpersonal exchanges may contribute to the re-proposal of that specific (positive) style in future relationships (Mannarini et al., 2021). On the contrary, caregivers presenting high levels of dysfunctional relational style (i.e., AA and/or RR) may be experienced by children as traumatic and such traumatic relational styles may be perpetrated by them in their own adult relationships (Cusinato, 2013). Experiencing an abusive and violent caregiver (i.e., AA and RR styles) may contribute to the reenactment of a violent relational style in future relationships (Mannarini et al., 2021).

The mechanisms underlying the intergenerational transmission of relational styles may be related to the fact that the experience of specific parental relational styles may lead children to learn that style as an appropriate strategy (Festinger and Carlsmith, 1959; Bandura, 1977, 1979; Stith et al., 2000; Kwong et al., 2003; Tyler et al., 2011; Cusinato, 2013). On the one hand, when caregivers interact according to the functional relational styles (i.e., CC), they share intense affect, they are nurturing and supporting, and they make children feel accepted and loved. These benevolent experiences contribute to the introjection of said positive relational styles and thus to the possible manifestation of children of warmth and flexibility in future relationships (L'abate, 1983; L'Abate et al., 2010). On the other hand, it has been shown that children who experienced dysfunctional parental styles, including corporal punishments, regardless of the frequency, learn these strategies as adequate and will repropose them in their adult relationships (Deater-Deckard et al., 2003; L'Abate et al., 2010). As a consequence, this may lead these children to learn from their parents to use violence as a strategy to handle quarrels with their partners or augmenting the possibility they may endure this strategy in future relationships (Kwong et al., 2003; L'Abate, 2005; L'Abate et al., 2010).

The RCT might be misunderstood as “an old wine in a new bottle” since it seems to present theoretical convergences with two other important theories, namely, attachment and social learning theories (Bowlby, 1973; Bandura, 1977; L'Abate et al., 2000, 2010; L'Abate, 2009). In fact, the literature shows that RCT and attachment theory, “*even though they derive from different theoretical sources, predict seemingly similar patterns of relationships between partners or among intimates*” (L'Abate et al., 2000, p. 104). However, differently from the attachment theory, the relational competence is specifically related to how we interact with others. Consequently, the identification of the relational competence of patients might give clinicians information on the ways of patients, either functional or dysfunctional, of relating to others and the strategies they more often implement in interpersonal exchanges. Regarding the intergenerational transmission of violence, both approaches emphasize the subjective experience relating to the parent-child relationship (McClellan and Killeen, 2000; L'Abate, 2005). However, the main difference between these two theories is the emphasis that the attachment theory gives to internal working models (IWMs). IWMs correspond to the mental representations of expectations from the self, others, and interpersonal relationships (Pietromonaco and Barrett, 2000). According to the attachment theory, early experiences of family abuse contribute to the development of IWMs that include violence with attachment figures, thus affecting the security of the attachment of individuals (Zeanah and Zeanah, 1989; McClellan and Killeen, 2000).

In contrast, RCT seems to present convergences and divergences with social learning theory (Bandura, 1977). In fact, both these approaches highlight the influence that the experience of a specific behavior might have on learning that conduct and use it in adult relationships (Bandura, 1977; L'Abate et al., 2010). According to the study by Bandura (1977), individuals can develop prosocial and criminal behaviors through the observation of the attitude of other people, their imitation, and the reinforcement of the behaviors apprehended (Wareham et al., 2009; Black et al., 2010). Therefore, the experience of family violence may teach offspring the appropriateness of that way of interacting with others through reinforcement (Black et al., 2010). One of the main differences between these two approaches is that, in the RCT, there is no mention of the concept of reinforcement, which instead plays a key role in the social learning theory.

## Implications for Clinical Practice

A better comprehension of the intergenerational transmission of relational styles could lead to several clinical implications. First, identifying both the relational styles patients experienced in childhood and the ones they act out in adult relationships may contribute to acquire information regarding the type of psychological interventions. For example, the AA style seems to be the most dysfunctional one as well as the most difficult one to change (L'Abate, 2005; L'Abate et al., 2010); thus, it requires a more structured type of psychological intervention. Second, the psychological interventions should also consider the interplay between psychological constructs and features that could have a role in the intergenerational transmission of relational styles, for instance, difficulties related to the emotional sphere (e.g., alexithymia and hyperarousal) (Gatta et al., 2017), self-awareness, and selfhood (i.e., the sense of self-importance experienced with own Self of an individual and others) (L'Abate, 2005; Gianesini, 2012). Third, clinical interventions should also consider psychological constructs that were already been demonstrated having an intergenerational transmission such as parental bonding, namely, the representation that children develop the contribution of caregivers to their relationship (Canetti et al., 2008; Balottin et al., 2017b). In addition, practitioners may also take into account the degree of self-awareness patients who have the relational styles of their own and others (Mannarini, 2009; Mannarini et al., 2016) and their perceptions of the Self (L'Abate, 2005; Whiting et al., 2009; Mannarini, 2010; Mannarini et al., 2013b; Mannarini and Boffo, 2014; Balottin et al., 2017a). Furthermore, clinicians should consider how patients describe the relational competences and styles of their parents, the impact that these had on their way of relating, and the meanings they give to these experiences (Gatta et al., 2016; Faccio et al., 2019).

Protective factors should be also targeted in the clinical practice. The self-importance of patients, which, according to RCT, corresponds to the importance a person attributes to the Self, represents a core factor in this regard (L'Abate, 2005; Whiting et al., 2009). In fact, it could be addressed by fostering the identification of all the practices through which a person can make an emotional investment in the Self and in other people (Lieberman et al., 2005). Another important protective factor could be the identification of benevolent early memories about relational experiences since they can contribute to the feeling of patients of self-importance and to counterbalance the scars of past negative relational experiences (Lieberman et al., 2005; L'Abate et al., 2010).

These considerations could help clinicians to plan *ad hoc* psychological interventions targeting the relational competence and styles of patients (L'Abate et al., 2010; Mannarini and Boffo, 2013; Mannarini et al., 2013a), with the two-fold aim to improve positive relational competences (i.e., CC) and to reduce dysfunctional relational styles (i.e., AA and/or RR).

## Discussion

Overall, according to the intergenerational transmission of relational styles (Cusinato, 2013), children could internalize the ways of interaction of parents with others, and they may use them in their own adult relationships (Roitman, 2017).

Moreover, it is important to highlight that recent research stressed out to employ a bidirectional approach to intergenerational transmission according to which caregivers and children influence each other in a reciprocal way (De Mol et al., 2013). Consequently, caregivers may socialize their offspring, and children may also socialize their parents (Parkin and Kuczynski, 2012; De Mol et al., 2013). Another feature that is important to highlight is that the intergenerational transmission does not imply a causal relationship: the experience of dysfunctional relational styles does not necessarily entail that the children will reenact similar conducts (Cusinato, 2013; Lünnemann et al., 2019). In fact, such experience represents only one of the factors (e.g., low level of education and poverty) that may contribute to the possible manifestation of the style experienced (Whiting et al., 2009; Franklin and Kercher, 2012; Cusinato, 2013). However, the mechanisms underlying the intergenerational transmission of relational styles are complex and need to be investigated further in future studies (L'Abate, 2005). In fact, despite the RCT was the main theory discussed earlier, future research should investigate the intergenerational transmission of relational styles by also considering and/or integrating different theories, which may provide a more complete explanation of the phenomenon.

The intergenerational transmission could have implications on both the research and the clinical level. In fact, the RCT gives importance to intimate relationships with others, as central elements for the development of personality and relational styles; for this reason, it could be useful in couple and family therapies (L'Abate, 1997).

Moreover, RCT allows a better understanding of the influence of relational competence and styles for the sense of Self of patients, significant relationships, and symptoms, thus, weighting them in psychological interventions. In addition, future studies should investigate the relationship between attachment and relational competence. In fact, if this relationship is proven, when knowing how individuals interact with others, it may be possible to have information on the attachment of individuals as well.

In conclusion, future studies should deepen both the intergenerational transmission of relational competence and styles and all the protective factors that may buffer the transmission of dysfunctional relational styles across generations.

## Author Contributions

FT and AAR conceived and wrote the manuscript. SM provided important intellectual contribution and critically revised the manuscript. All authors approved the final version.

## Conflict of Interest

The authors declare that the research was conducted in the absence of any commercial or financial relationships that could be construed as a potential conflict of interest.

## Publisher's Note

All claims expressed in this article are solely those of the authors and do not necessarily represent those of their affiliated organizations, or those of the publisher, the editors and the reviewers. Any product that may be evaluated in this article, or claim that may be made by its manufacturer, is not guaranteed or endorsed by the publisher.
